# Application of a Biologically Contained Reporter System To Study Gain-of-Function H5N1 Influenza A Viruses with Pandemic Potential

**DOI:** 10.1128/mSphere.00423-20

**Published:** 2020-08-26

**Authors:** Eva E. Spieler, Eva Moritz, Silke Stertz, Benjamin G. Hale

**Affiliations:** a Institute of Medical Virology, University of Zurich, Zurich, Switzerland; b Life Science Zurich Graduate School, ETH and University of Zurich, Zurich, Switzerland; University of Pittsburgh School of Medicine

**Keywords:** H5N1, antiviral agents, avian viruses, biological containment, biosafety, gain of function, influenza, reporter genes

## Abstract

Natural adaptation of an antigenically novel avian influenza A virus (IAV) to be transmitted efficiently in humans has the potential to trigger a devastating pandemic. Understanding viral genetic determinants underlying adaptation is therefore critical for pandemic preparedness, as the knowledge gained enhances surveillance and eradication efforts, prepandemic vaccine design, and efficacy assessment of antivirals. However, this work has risks, as making gain-of-function substitutions in fully infectious IAVs may create a pathogen with pandemic potential. Thus, such experiments must be tightly controlled through physical and biological risk mitigation strategies. Here, we applied a previously described biological containment system for IAVs to a 2009 pandemic H1N1 strain and a highly pathogenic H5N1 strain. The system relies on deletion of the essential viral hemagglutinin (HA) gene, which is instead provided in *trans*, thereby restricting multicycle virus replication to genetically modified HA-complementing cells. In place of HA, a *Renilla* luciferase gene is inserted within the viral genome, and a live-cell luciferase substrate allows real-time quantitative monitoring of viral replication kinetics with a high dynamic range. We demonstrate that biologically contained IAV-like particles exhibit wild-type sensitivities to approved antivirals, including oseltamivir, zanamivir, and baloxavir. Furthermore, the inability of these IAV-like particles to genetically acquire the host-encoded HA allowed us to introduce gain-of-function substitutions in the H5 HA gene that promote mammalian transmissibility. Biologically contained “transmissible” H5N1 IAV-like particles exhibited wild-type sensitivities to approved antivirals, to the fusion inhibitor S20, and to neutralization by existing H5 monoclonal and polyclonal sera. This work represents a proof of principle that biologically contained IAV systems can be used to safely conduct selected gain-of-function experiments.

**IMPORTANCE** Understanding how animal influenza viruses can adapt to spread in humans is critical to prepare for, and prevent, new pandemics. However, working safely with pathogens that have pandemic potential requires tight regulation and the use of high-level physical and biological risk mitigation strategies to stop accidental loss of containment. Here, we used a biological containment system for influenza viruses to study strains with pandemic potential. The system relies on deletion of the essential HA gene from the viral genome and its provision by a genetically modified cell line, to which virus propagation is therefore restricted. We show that this method permits safe handling of these pathogens, including gain-of-function variants, without the risk of generating fully infectious viruses. Furthermore, we demonstrate that this system can be used to assess virus sensitivity to both approved and experimental drugs, as well as the antigenic profile of viruses, important considerations for evaluating prepandemic vaccine and antiviral strategies.

## Abstract (precis)

Understanding how animal influenza viruses can adapt to spread in humans is critical to prepare for, and prevent, new pandemics. However, working safely with pathogens that have pandemic potential requires tight regulation and the use of high-level physical and biological risk mitigation strategies to stop accidental loss of containment. Here, we used a biological containment system for influenza viruses to study strains with pandemic potential. The system relies on deletion of the essential HA gene from the viral genome and its provision by a genetically modified cell line, to which virus propagation is therefore restricted. We show that this method permits safe handling of these pathogens, including gain-of-function variants, without the risk of generating fully infectious viruses. Furthermore, we demonstrate that this system can be used to assess virus sensitivity to both approved and experimental drugs, as well as the antigenic profile of viruses, important considerations for evaluating prepandemic vaccine and antiviral strategies.

## INTRODUCTION

Seasonal influenza A viruses (IAV) can cause an acute respiratory illness in humans, with symptoms including fever, dry cough, headache, muscle pain, and malaise. The disease is typically mild and self-resolving, but it can be more severe and lead to death, particularly in high-risk groups, such as young children, the elderly, and those with chronic underlying health conditions or an immunosuppressed state. Therefore, annual seasonal IAV epidemics remain a major threat to public health and are associated with significant morbidity and mortality worldwide, including an estimated 290,000 to 650,000 deaths globally (1). Moreover, IAVs found in nonhuman hosts (e.g., avian reservoirs) have the potential for sporadic zoonotic transmission to humans and can cause critical disease, such as severe pneumonia, acute respiratory distress syndrome, and significant fatalities, even in young, otherwise healthy adults (2). For example, avian H5N1 IAVs have caused 861 recorded human infections and 455 deaths since 2003, while avian H7N9 IAVs have caused >1,500 human infections since 2013 (2). The fear is that one of these novel avian IAV strains will eventually adapt to replicate and transmit efficiently in humans, thereby sparking a devastating new influenza pandemic similar in scale to one of the three that occurred in the 20th century: the 1918 H1N1 pandemic, with >40 million deaths; the 1957 H2N2 pandemic, with 1 to 3 million deaths; and the 1968 H3N2 pandemic, with ∼1 million deaths (3–7).

For pandemic preparedness, it is important to understand the genetic determinants of IAVs that permit them to replicate, to be transmitted, and to cause disease in humans. Such work allows the identification of sequence markers that can be used in global surveillance and eradication efforts, and it supports testing of prepandemic vaccine and antiviral stockpiles to ensure their efficacy if novel IAV strains acquire human replication and transmissibility features (8, 9). Key landmark studies in this area identified several amino acid substitutions in viral proteins, notably hemagglutinin (HA), that are critical for allowing mammalian transmissibility of avian IAVs, and identification was facilitated by the creation of fully infectious, replication-competent, and antigenically novel IAVs at the highest biosafety and biosecurity levels (10–13). The creation of and work with such novel, transmissible IAVs has been controversial in the scientific community, leading to heated debates in the literature over the risk-benefit analysis of so-called gain-of-function experiments (14–32), research moratoria on such pathogens (33–36), and discussion about the policy development of new biocontainment, biosafety, and biosecurity frameworks (37–41).

Biologically contained viruses, where an essential viral gene is deleted from a pathogen and is expressed in *trans* by a complementing cell line to allow propagation, represent a safe means to study fundamental *in vitro* aspects of pathogen biology and could constitute a risk mitigation strategy for some gain-of-function experiments. Biological containment methods have previously been applied to study highly pathogenic viruses such as Ebola virus and Omsk hemorrhagic fever virus safely at lower containment levels than their usual biosafety level 4 (BSL4) facility requirements (42, 43), thereby opening up a range of valuable experimental activities due to the lower associated operating costs, increased equipment availability, and number of laboratories that can use them. Often, in these methods, the essential viral gene is replaced with a convenient reporter gene, such as one encoding a fluorescent protein or luciferase, in order to simplify subsequent studies such as high-throughput screening of large compound libraries with antiviral potential.

Recent efforts in the development of biologically contained IAVs for different applications have been reviewed extensively (44). Most previous studies have focused on using common laboratory IAV strains, such as PR8 or WSN, and studying concepts such as (bivalent) vaccine or vector design, drug resistance or screening, host factor dependencies, and RNA packaging. In this study, we sought to assess the applicability of a biologically contained IAV concept, with viral deletion of HA (45), to strains such as highly pathogenic H5N1 and subsequently derived gain-of-function mammalian-transmissible variants that would otherwise require BSL3 or higher biocontainment facilities. Our specific aim was to understand the feasibility of using these tools as a safe risk mitigation strategy to assess antiviral drug susceptibility and antigenicity of new variants without having to generate fully infectious, replication-competent IAVs.

## RESULTS

### Generation of biologically contained 2009 pandemic H1N1 and highly pathogenic H5N1 IAVs expressing *Renilla* luciferase.

**FIG 1:**
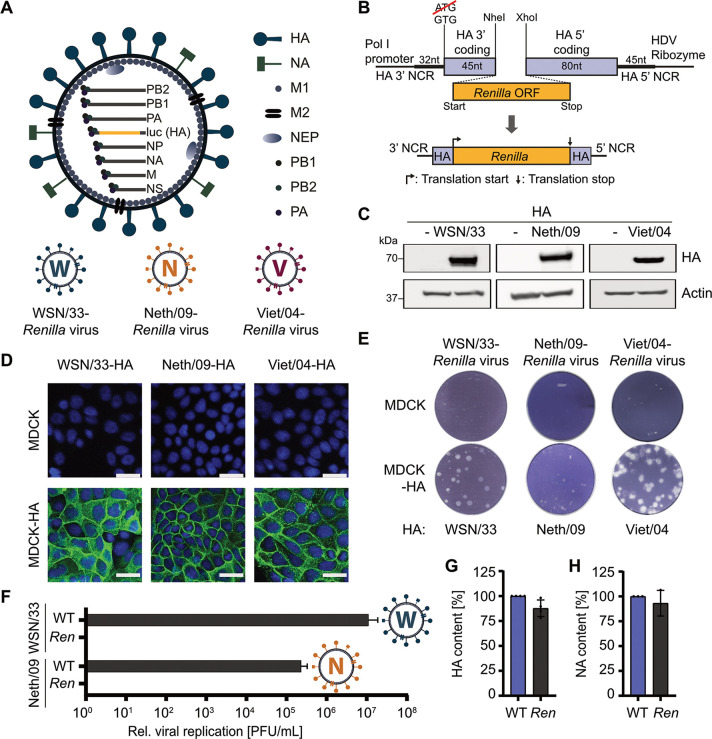
Biologically contained WSN/33, 2009 pandemic H1N1, and highly pathogenic H5N1 IAVs expressing *Renilla* luciferase. (A and B) Schematic representations of the reporter virus strategy. The HA segment is modified to encode *Renilla* luciferase instead of the glycoprotein HA but retains specific WSN/33 HA segment noncoding and coding packaging sequences, as shown. The original HA start codon is mutated to prevent erroneous translation. All other segments are fully derived from the respective IAV strain: WSN/33 (H1N1) (46), A/Netherlands/602/09 (Neth/09; pdmH1N1), or A/VietNam/1203/04 (Viet/04; H5N1). (C) Western blot analysis of MDCK and MDCK-HA cell lines stably expressing each strain-specific HA. Specific antibodies were used to detect HA and actin. (D) Indirect immunofluorescence analysis of MDCK and MDCK-HA cell lines stably expressing each strain-specific HA (green). DAPI was used to stain cell nuclei (blue). Bars, 25 μm. (E) Plaque formation assay for each IAV reporter on MDCK and MDCK-HA cell lines stably expressing each strain-specific HA. Each cell line was infected with the same number of PFU, and assays were fixed and stained 36 h later. (F) Passaging of WSN/33 and WSN/33-*Renilla* IAV particles, as well as Neth/09 and Neth/09-*Renilla* IAV particles, confirms biological containment of the IAV reporters. Data are means and standard deviations of final virus titers on MDCK cells (*n* = 3) following three blind passages on MDCK cells (i.e., transferring half the supernatant to fresh cells every 48 h) after an initial infection at an MOI of 0.001 PFU/cell. (G) Relative levels of HA, as determined by quantitative Western blotting of equal PFU of WSN/33 and WSN/33-*Renilla* IAVs. Data are means and standard deviations from four independent Western blots and were normalized to WSN/33 levels. (H) Relative levels of NA between equal PFU of WSN/33 and WSN/33-*Renilla* IAVs, as determined by an NA-Star influenza virus neuraminidase inhibitor resistance detection kit. Values from three independent replicates, each performed in duplicate, were normalized to the value for WSN/33. Data are means and standard deviations.

Previous studies have described the construction of a recombinant IAV (A/WSN/33 strain [referred to here as WSN]) in which the HA coding sequence is replaced with sequences for either green fluorescent protein (GFP) or *Renilla* luciferase, but 3′ and 5′ packaging regions of the HA segment are maintained (45, 46). In MDCK cells engineered to stably express WSN HA, such reporter IAV-like particles can undergo multicycle replication (45) but are restricted to single-cycle infections in cells lacking expression of a complementing HA molecule (46). While WSN is a commonly used strain in many laboratories because of its high-growth properties and has been used to identify important aspects of IAV replication, we sought to expand use of this technology to more clinically relevant strains, such as 2009 pandemic H1N1 (pdmH1N1) and highly pathogenic H5N1 IAVs (Fig. 1A and B). To this end, we generated MDCK cell lines stably expressing either the HA protein from pdmH1N1 (strain A/Netherlands/602/2009 [Neth/09]) or a modified HA protein from H5N1 (A/VietNam/1203/04 [Viet/04]), which lacks the polybasic cleavage site (termed HALo) (47), and verified their stable expression by Western blotting (Fig. 1C). Indirect immunofluorescence also revealed that each engineered MDCK cell line exhibited homogenous expression of the respective HA and that all HAs predominantly localized to the cell surface (Fig. 1D). Using these cell lines, we were able to successfully rescue and grow recombinant Neth/09-*Renilla* and Viet/04-*Renilla* IAV-like particles that incorporate a segment expressing *Renilla* luciferase and lack the coding and noncoding sequences for their own HAs but are otherwise authentic. Importantly, these Neth/09-*Renilla* and Viet/04-*Renilla* IAV-like particles, like the original WSN/33-*Renilla* IAV, are biologically contained, as they do not undergo multicycle replication to form plaques on MDCK cells but readily form plaques in MDCK cells stably expressing the cognate HA (Fig. 1E). In addition, passaging experiments confirmed that biologically contained IAV-like particles do not propagate in MDCK cells even to low levels (Fig. 1F), and functional experiments indicated that the IAV-like particles (at least for the WSN strain) can have levels of HA and NA similar to those of authentic virus particles (Fig. 1G and H).

### Biologically contained IAV reporters expressing *Renilla* luciferase allow live-cell kinetic analysis of single-cycle and multicycle infections.

**FIG 2:**
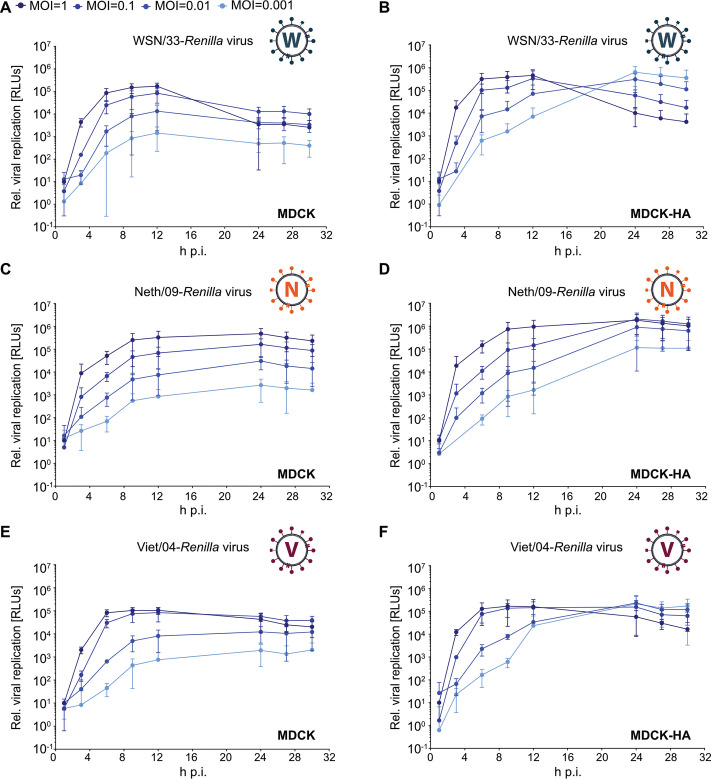
Live-cell kinetic analysis of single-cycle and multicycle infections with the biologically contained IAV reporters expressing *Renilla* luciferase. MDCK cells (A, C, and E) or MDCK-HA cells (B, D, and F) in a 96-well format were infected with the indicated IAV reporter viruses at different MOI (1, 0.1, 0.01, or 0.001 PFU/cell) or mock infected. After 1 h adsorption, EnduRen substrate was added to the overlay medium, and luminescence (in relative light units [RLUs]) was determined directly in live cells every 3 h and normalized to the value from mock-infection conditions. Mean values from three independent experiments are plotted, with error bars representing standard deviations.

*Renilla* luciferase was chosen as our reporter molecule because of two key advantages over other commonly used reporters, such as fluorophores and the small luciferase NanoLuc: (i) luciferases in general have higher dynamic ranges than fluorophores, such as GFP, allowing more optimal discrimination between small interventions (48), and (ii) a unique, stable, live-cell substrate system is available for *Renilla* luciferase (EnduRen; Promega), which means that cell lysis or supernatant collection is unnecessary, minimizing the liquid handling required with most other luminescent substrates and allowing simple continuous live-cell monitoring over long periods with multiple time points. To demonstrate this system, we infected MDCK or MDCK-HA cells with either WSN/33-*Renilla*, Neth/09-*Renilla*, or Viet/04-*Renilla* IAV-like particles at a range of multiplicities of infection (MOI) and added EnduRen substrate to the medium at 1 h postinfection. With no other manipulation, we were able to perform direct live-cell luminescence readouts every 3 h until the viral replication kinetics were complete (Fig. 2A to F). Notably, in preliminary experiments we found that the EnduRen substrate is stable in tissue culture medium for at least 30 h and does not need to be replenished. Relatively high-MOI (1 and 0.1 PFU/cell) infections of MDCK or MDCK-HA cells with all of the reporter IAVs led to a rapid increase in luminescence emission, peaking at 6 to 9 h postinfection and giving an ∼1,000-fold increase of activity over mock infections. No differences in these replication kinetics or magnitude were obvious between experiments in MDCK cells with and without complementing HA expression (Fig. 2A to F). In contrast, relatively low-MOI (0.01 and 0.001 PFU/cell) infections showed peak luminescence levels only ∼10- to 100-fold above that of mock infection in MDCK cells lacking HA expression (Fig. 2A, C, and E), suggesting only single-cycle infections in these cells, but led to luminescence levels ∼1,000-fold higher than mock infection at later time points (12 to 24 h postinfection) in MDCK cells expressing HA, presumably due to multicycle replication in these cells (Fig. 2B, D, and F). These data indicate that the IAV-*Renilla* reporters described here are biologically contained and can be effectively used to study single-cycle and multicycle replication kinetics under live-cell conditions with a very high dynamic range.

### Biologically contained IAV reporters exhibit antiviral drug sensitivities similar to those of authentic IAVs.

**FIG 3:**
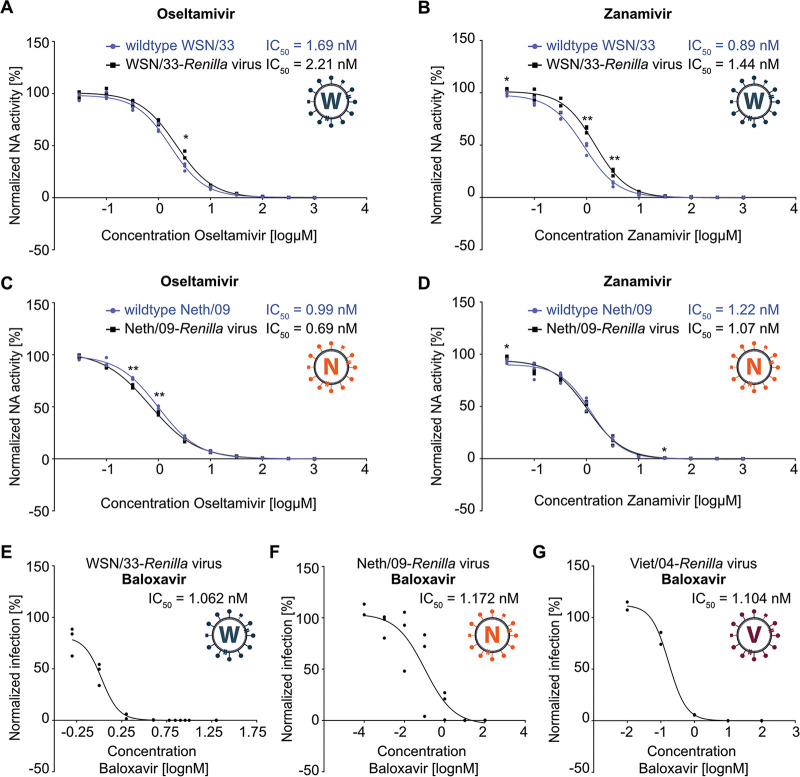
Biologically contained IAV reporters exhibit antiviral drug sensitivities similar to those of authentic IAVs. Oseltamivir and zanamivir sensitivity of WSN/33 and WSN/33-*Renilla* IAV particles (A and B) or Neth/09 and Neth/09-*Renilla* IAV particles (C and D) was measured using the NA-Star influenza virus neuraminidase inhibitor resistance detection kit. Values from three independent replicates, each performed in duplicate, were normalized to the respective untreated control, plotted, and used to calculate IC_50_s with GraphPad Prism 7.05. The data line represents the best-fit curve. Statistical significance was determined for each concentration by unpaired two-tailed *t* test and marked where thresholds were reached (*, *P < *0.05; **, *P < *0.01). (E to G) MDCK-HA cells expressing the respective HA were infected with WSN/33-*Renilla* IAV, Neth/09-*Renilla* IAV, or Viet/04-*Renilla* IAV and treated with different concentrations of baloxavir, together with the EnduRen substrate, at 1 h postinfection. Luminescence (in RLUs) was determined directly in live cells every 3 h and normalized to the value obtained under mock-infection conditions. Area-under-the-curve (AUC) values from three independent experiments were plotted and used to calculate IC_50_s with GraphPad Prism 7.05. The data line represents the best-fit curve.

To evaluate the feasibility of using biologically contained IAV reporters for inhibitor studies, we assayed their sensitivity to known, approved antiviral drugs such as oseltamivir, zanamivir, and baloxavir. Oseltamivir and zanamivir are approved neuraminidase (NA) inhibitors (4). Using standard *in vitro* NA inhibition assays, we determined that both oseltamivir and zanamivir potently inhibit the NA activity of WSN/33-*Renilla* IAV-like particles with a 50% inhibitory concentration (IC_50_) similar to that required for inhibition of authentic WSN IAV particles (Fig. 3A and B). The NA activity of Neth/09-*Renilla* IAV-like particles was also inhibited to the same extent as wild-type Neth/09 IAV particles by these two antivirals (Fig. 3C and D). These data indicate that the biologically contained IAV reporters can be used to faithfully recapitulate inhibitor study data of authentic viruses, making them a useful surrogate when work with fully infectious IAVs has to be limited. We further profiled the antiviral activity of baloxavir, a potent inhibitor of the viral endonuclease (49), against all of our IAV-*Renilla* reporters. MDCK cells expressing each cognate HA were infected with the respective IAV-*Renilla* virus in the presence of various concentrations of baloxavir. Following addition of EnduRen to the medium at 1 h postinfection, together with baloxavir, direct live-cell luminescence readouts were taken at several time points postinfection, and IC_50_s were calculated from the nonlinear regression curve that was determined for each virus from the area under the curve (AUC) of the replication kinetics (Fig. 3E to G). Baloxavir was able to potently interfere with the replication of all three *Renilla* reporter viruses (WSN/33, Neth/09, and Viet/04 based), with IC_50_s in the range of those published previously (50, 51). Thus, biologically contained IAV reporters can also be used to rapidly assess antiviral efficacy without laborious and time-consuming titrations of the wild-type viruses.

### Generation of a biologically contained IAV reporter system to study mammalian-transmissible gain-of-function H5N1 variants.

**FIG 4:**
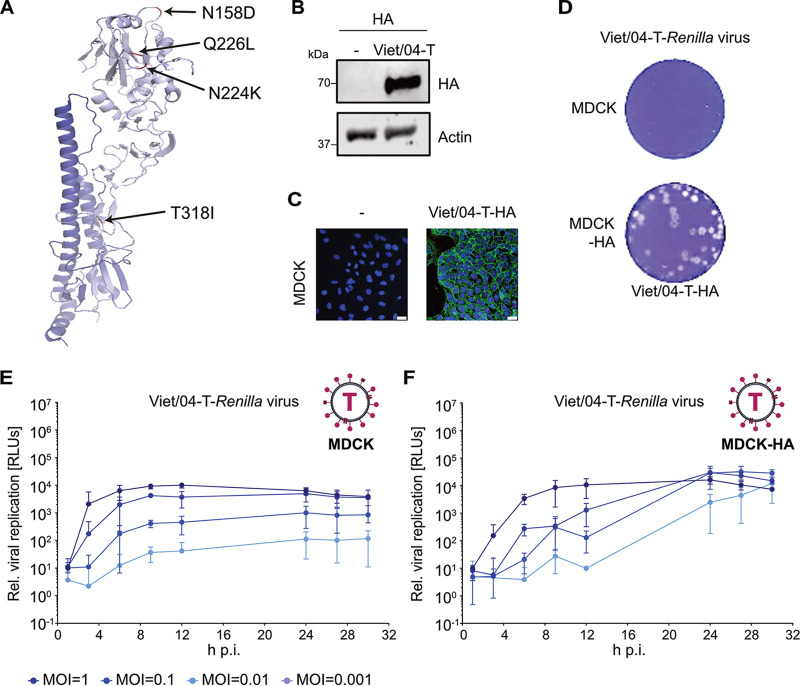
Generation of a biologically contained H5N1 IAV reporter with gain-of-function HA amino acid substitutions. (A) Previously described crystal structure of the transmissible Viet/04 HA mutant (PDB ID: 4N5Z) highlighting the four amino acid substitutions that confer mammalian transmissibility to H5 HA (H3 numbering) (53). (B) Western blot analysis of MDCK cells stably expressing Viet/04-T-HA and control MDCK cells. Specific antibodies were used to detect HA and actin. (C) Indirect immunofluorescence analysis of the above-mentioned cell lines. HA is stained green, while DAPI was used to stain cell nuclei (blue). Bars, 25 μm. (D) Plaque formation assay for the Viet/04-T-*Renilla* IAV on MDCK and MDCK-Viet/04-T-HA cell lines. Each cell line was infected with the same number of PFU, and assays were fixed and stained 36 h later. (E and F) MDCK cells (E) or MDCK-Viet/04-T-HA cells (F) in a 96-well format were infected with Viet/04-T-*Renilla* IAV at different MOI (1, 0.1, 0.01, or 0.001 PFU/cell) or mock infected. After 1 h adsorption, EnduRen substrate was added to the overlay medium, and luminescence (in RLUs) was determined directly in live cells every 3 h and normalized to the value obtained under mock-infection conditions. Mean values from three independent experiments are plotted, with error bars representing standard deviations.

Following the discovery that highly pathogenic H5N1 IAVs can be experimentally adapted in their HA sequence to confer mammal-to-mammal transmissibility (10, 11), there has been a detailed discussion in the field about the biosafety, biosecurity, and ethical implications of studying such gain-of-function viruses (14–32). To overcome some of these barriers and concerns, biological risk mitigation strategies are an additional layer of molecular safety to enhance physical biocontainment (BSL3+) infrastructure and protocols. For example, genetic engineering of species-specific microRNA target sites into viral genomes can limit replication exclusively to nonhuman experimental models (52). As proof of principle for another biological risk mitigation strategy, we applied the biologically contained IAV reporter system described here to this problem. Although such a system limits experiments to *in vitro* cell culture work, we reasoned that a major advantage would be the sensitive reporter read-out at a lower physical biocontainment level (e.g., BSL2). Importantly, in such a system, there is no mechanism for the virus to genetically acquire the variant HA conferring transmissibility, as the HA cDNA provided in *trans* lacks essential 3′ and 5′ noncoding packaging sequences. We also used a human codon-optimized HA sequence to rule out the very rare chance of recombination. As before, and primarily for added biosafety in these proof-of-principle experiments, the construct was based on the modified H5 HA protein lacking the polybasic cleavage site (HALo), although it should be possible to use an HA containing the polybasic cleavage site. We thus generated an MDCK cell line stably expressing a variant Viet/04 H5 HA (HALo) protein with 4 amino acid substitutions described to confer airborne transmissibility (termed Viet/04-T-HA): N158D, N224K, Q226L, and T318I (H3 numbering) (Fig. 4A) (11, 53). The two substitutions in the HA globular head (N224K and Q226L), together with loss of the glycosylation site at position 158 (N158D), are responsible for increasing the affinity of HA for human-type receptors (11, 53–56). In addition, the T318I substitution increases HA thermostability and lowers the pH required for fusion (11, 53, 57). Stable homogenous expression and correct localization of the Viet/04-T-HA protein in MDCK cells was confirmed by Western blotting and indirect immunofluorescence (Fig. 4B and C). We grew a stock of Viet/04-*Renilla* IAV on these cells to produce virus-like particles incorporating the Viet/04-T-HA protein and confirmed that this new Viet/04-T-*Renilla* IAV is biologically contained: it forms plaques and undergoes multicycle replication, as determined by luciferase activity, only on MDCK cells that stably express HA (Fig. 4D to F). These data indicate that a biologically contained H5N1 reporter virus with gain-of-function mutations that confer mammalian transmissibility can be produced and used for facile assays.

### The biologically contained mammalian-transmissible H5N1 reporter IAV can be used to assay antiviral-drug sensitivity and antigenicity.

**FIG 5:**
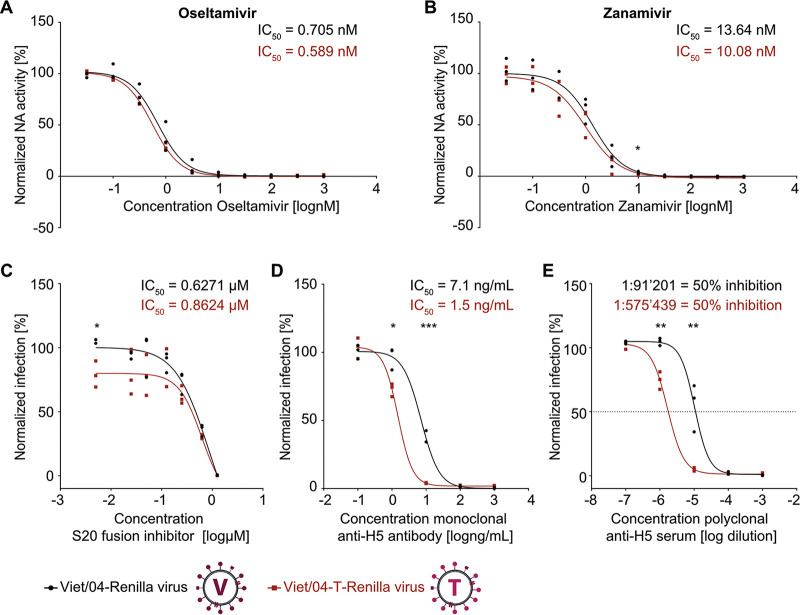
Antiviral drug sensitivity and antigenicity of a biologically contained H5N1 IAV reporter with gain-of-function HA amino acid substitutions. Oseltamivir carboxylate and zanamivir sensitivity of Viet/04-*Renilla* and Viet/04-T-*Renilla* IAV particles (A and B) was measured using the NA-Star influenza virus neuraminidase inhibitor resistance detection kit. Values from three independent replicates, each performed in duplicates, were normalized to the respective untreated control, plotted, and used to calculate IC_50_s with GraphPad Prism 7.05. The data line represents the best-fit curve. (C) MDCK cells were infected with Viet/04-*Renilla* or Viet/04-T-*Renilla* IAV at an MOI of 0.5 PFU/cell and different concentrations of the S20 fusion inhibitor were added together with the EnduRen substrate. Luminescence (in RLUs) was determined directly in live cells every 3 h and normalized to the value obtained under mock-infection conditions. Area-under-the-curve (AUC) values from three independent experiments were plotted and used to calculate IC_50_s with GraphPad Prism 7.05. The data line represents the best-fit curve. (D and E) A monoclonal antibody (D) or a polyclonal serum (E) specific for H5 HA was serially diluted and preincubated with fixed amounts of Viet/04-*Renilla* or Viet/04-T-*Renilla* IAV for 1 h prior to infection of MDCK cells and addition of EnduRen substrate. Luminescence (in RLUs) was determined directly in live cells every 3 h and normalized to the value obtained under mock-infection conditions. AUC values from three independent experiments were plotted and used to calculate IC_50_s (D) or the dilution factor for 50% inhibition (E) with GraphPad Prism 7.05. The data line represents the best-fit curve. For all panels, statistical significance was determined for each concentration by an unpaired two-tailed *t* test and marked where thresholds were reached (*, *P < *0.05; **, *P < *0.01; ***, *P < *0.001).

As a proof of concept for studying mammalian-transmissible gain-of-function IAV-like particles under biologically contained conditions, we assayed how the substitutions in Viet/04-T-HA impacted the effect of antivirals and antibodies. Standard *in vitro* NA inhibition assays revealed that both oseltamivir and zanamivir inhibit the NA activity of Viet/04-T-*Renilla* IAV-like particles with IC_50_s similar to those required for inhibition of Viet/04-*Renilla* IAV-like particles, demonstrating similar incorporation of NA into both viruses (Fig. 5A and B). We also found that, despite the reported impact of the T318I substitution on HA thermostability and fusion potential (11, 53, 57), the potent experimental HA-targeting fusion inhibitor S20 (58) is as active against Viet/04-T-*Renilla* IAV as it is against Viet/04-*Renilla* IAV during infection (Fig. 5C). In addition, we tested the neutralization capabilities of a monoclonal anti-H5 HA antibody and a polyclonal anti-H5 HA serum, with the aim of assessing whether the 4 gain-of-function amino acid substitutions impacted the antigenic profile of HA, an important consideration for development of prepandemic H5 vaccines. Viet/04-T-*Renilla* IAV-like and Viet/04-*Renilla* IAV-like particles were preincubated for 1 h with each antibody or serum at various concentrations prior to single-cycle infection of MDCK cells. Following addition of EnduRen, luciferase activity was measured at several times postinfection, and IC_50_s, or dilution factors with 50% inhibition, were calculated using the nonlinear regression curve for each infection (Fig. 5D and E). Importantly, Viet/04-T-*Renilla* IAV was sensitive to neutralization by both the anti-H5 monoclonal and polyclonal preparations and was, if anything, more sensitive to neutralization than Viet/04-*Renilla* IAV. These data show that the biologically contained IAV reporter system described here can be applied to safely study gain-of-function mutations, and assays can be readily established to understand antiviral susceptibility and potential antigenic changes.

## DISCUSSION

Herein, we describe the application of a biologically contained IAV reporter system to clinically relevant strains, including 2009 pdmH1N1 and highly pathogenic H5N1. We show that by incorporating *Renilla* luciferase as the reporter of choice, and by using a stable, cell-permeable substrate (EnduRen), the high dynamic range of luciferase can be combined with real-time live-cell kinetic measurements to track virus replication in a single sample without excessive liquid-handling steps. The biologically contained system relies on the in *trans* expression of HA in a complementing cell line to achieve multicycle replication; thus, the use of appropriate cell lines allows ready comparisons between single-cycle and multicycle replication. Furthermore, we demonstrate that these systems are fully amenable to assessment of antiviral drug sensitivity, with inhibitory effects aligning well with those of wild-type nonreporter viruses. Given that these safe, clinically relevant reporter viruses are already optimized for BSL2 use in multiwell formats, such as 96-well plates, they could be useful for conducting large-scale high-throughput compound screens to identify new antivirals.

The biological containment system used here permits the cell culture study of otherwise BSL3 or BSL3+ IAV strains at BSL2 without the risk of reassortment or recombination. In this regard, expression of HA in *trans*, with no possibility of its being genetically incorporated into a fully infectious virus, is the essential feature, as IAVs with novel HAs to which humans do not yet have any humoral immunity are the most threatening with regard to pandemic emergence (4). Such a strict genetic safety feature allowed us to create reporter H5N1 IAV variants utilizing gain-of-function mammalian-transmissible HA proteins that are replication competent only in cells stably expressing HA. So-called gain-of-function experiments are controversial for pathogens with pandemic potential (14–32), and it is important to implement risk mitigation strategies as much as possible wherever the scientific question under study allows. Here, we did not want to address the mammalian transmissibility or virulence of gain-of-function H5N1 IAVs (which would require fully replication competent viruses), but instead we sought to use the biologically contained reporter system as a proof of principle for developing a feasible framework to assess the antiviral susceptibility and antigenicity of such viruses. Given our success in establishing and implementing such a system, as well as our results indicating that amino acid substitutions related to mammalian transmissibility do not reduce H5N1 antiviral sensitivity or antigenicity, we believe that the biologically contained reporters used here represent a safe and useful way to characterize IAVs with pandemic potential.

## MATERIALS AND METHODS

### Cells, lentiviruses, and generation of cell lines.

Madin-Darby canine kidney (MDCK) and human embryonic kidney (293T) cells were grown in Dulbecco’s modified Eagle’s medium (DMEM) supplemented with 10% fetal bovine serum (FBS) and 1% penicillin-streptomycin (P/S) at 37°C with 5% CO_2_. To generate cell lines stably expressing HA, the respective HA cDNAs were first cloned into pLVX-IRES-Puro (Clontech Laboratories) using XhoI and BamHI (Viet/04) or XhoI and NotI (Neth/09 and WSN/33) restriction sites. HA cDNAs were obtained either from existing vectors (WSN/33, Neth/09, and Viet/04) or by commercial gene synthesis (Viet/04-T; GeneArt; Thermo Fisher Scientific). Both Viet/04-derived HA constructs lacked the sequence encoding the polybasic cleavage site (and were therefore termed HALo), and all constructs were validated by sequencing prior to use. To generate lentiviral particles, 293T cells were cotransfected with pCMVdR8.91, pMD2.G, and the respective pLVX-HA-IRES-Puro construct for 48 h before harvesting of supernatants, filtration through a 0.45-μm filter, and transduction of MDCK cells in the presence of Polybrene (final concentration of 8 μg/ml; Sigma-Aldrich). Two days later, transduced cells were selected with puromycin (3.5 μg/ml; Thermo Fisher Scientific) and subcloned by limiting dilution. Clonal cell colonies were expanded, and HA expression was assayed by Western blotting and immunofluorescence.

### SDS-PAGE and Western blotting.

Cell or virus lysates were prepared in 2× urea disruption buffer (6 M urea, 4% SDS, 1 M β-mercaptoethanol, bromophenol blue), sonicated, and heated to 95°C for 7 min. Proteins were separated by SDS-PAGE on 4 to 12% NuPAGE bis-Tris gradient gels (Life Technologies) and transferred to nitrocellulose membranes (GE Healthcare, Amersham). Proteins were detected using antibodies specific for WSN/33-HA (1:5) (59), Viet/04-HA (1:1,000; no. 200-301-976; Rockland), Neth/09-HA (1:500; 19D3 [60]), or β-actin (no. sc-47778; Santa Cruz). The secondary antibody used was a fluorochrome-conjugated anti-mouse immunoglobulin (no. 35519; Thermo Fisher Scientific). A Li-Cor Odyssey scanner was used for detection and quantification.

### Immunofluorescence.

Cells were seeded on glass coverslips in 24-well plates and the next day fixed with 3% paraformaldehyde in phosphate-buffered saline (PBS) for 15 min at room temperature (RT), followed by permeabilization with 0.5% Triton X-100 in PBS for 5 min. After blocking with 2% FBS for 1 h, staining was performed in 2% FBS for 1 h at RT using antibodies specific for WSN/33 HA (1:5) (59), Viet/04 HA (1:1,000; no. 200-301-976; Rockland), or Neth/09 HA (1:500; 19D3 [60]). The secondary antibodies used were Alexa Fluor-conjugated donkey anti-mouse and -rabbit IgG antibodies (no. A21202 and A21206; Life Technologies). Cell nuclei were stained with 4′,6′-diamidino-2-phenylindole (DAPI; 1:1,000), and images were analyzed using a Leica TCS SP5 laser scanning confocal microscope system.

### Production of biologically contained IAV-like particles expressing *Renilla* luciferase.

WSN/33-*Renilla* IAV has been described previously (46). Briefly, a pPolI-based plasmid (61) was constructed harboring the *Renilla* luciferase open reading frame (ORF) (REN) flanked by the WSN/33 HA 3′ and 5′ noncoding regions (32 and 45 nucleotides, respectively) and 45 (3′) and 80 (5′) nucleotides of the HA ORF packaging signals. The original HA ORF ATG (in positive sense) was mutated to GTG (pPolI-HA-REN-HA) (Fig. 1B). To generate Neth/09-*Renilla* and Viet/04-*Renilla* IAV-like particles, 293T cells were cotransfected with 7 ambisense pDZ plasmids encoding each segment of the respective IAV strain (PB2, PB1, PA, NP, NA, M, and NS but not HA), pPolI-HA-REN-HA, and a pCAGGS vector expressing the respective complementing HA protein. Plasmids to rescue recombinant Neth/09 IAVs have been described previously (62), and plasmids to rescue recombinant Viet/04 HALo IAVs were kindly provided by Adolfo García-Sastre, Icahn School of Medicine at Mount Sinai, New York, NY (47). Strain-matched MDCK-HA cells were coseeded together with the transfected 293T cells, and the medium was supplemented with 1 μg/ml tosylsulfonyl phenylalanyl chloromethyl ketone (TPCK)-treated trypsin (Sigma-Aldrich, MO). Forty-eight hours posttransfection, supernatants were harvested, single clones were plaque purified on fresh MDCK-HA cells, and virus stocks were grown and titrated using standard methods on the respective MDCK-HA cell line. To generate Viet/04-T-*Renilla* IAV-like particles, Viet/04-*Renilla* IAV was grown on the MDCK cell line expressing Viet/04-T-HA. All virus stocks were sequence verified by full genome sequencing using previously described protocols and primers (63, 64).

### Biosafety.

Procedures to generate and work with biologically contained H5N1 IAVs were thoroughly risk assessed prior to the start of the project and were approved by the Swiss Federal Office of Public Health (Ecogen number A161912). In brief, special considerations included the temporal and physical separation of experiments involving H5N1 IAV-like particles from experiments involving other IAVs in order to minimize the risk of accidental reassortment. With specific regard to the H5N1 IAV HA and its mammalian-transmissible variant, we did not at any point generate or propagate pDZ- or pPolI-based plasmids containing the respective full HA segment so as to prevent accidental rescue of replication-competent viruses containing the H5 HA segment: we used only plasmids carrying the HA ORF which lacked the 3′ and 5′ noncoding sequences essential for packaging. In addition, for the mammalian-transmissible H5 HA variant, we generated a human codon-optimized HA ORF lacking the 3′ and 5′ noncoding sequences. The rationale for this strategy was to destroy any potential cryptic packaging elements in the coding region, thereby eliminating the very rare chance of recombination of this sequence into a fully replication-competent virus.

### Assessing virus replication by live-cell *Renilla* luciferase assays.

Cells in 96-well plates were washed once with PBS prior to infections with the different viruses at the appropriate MOI in PBS supplemented with 1 mM Ca^2+^ and Mg^2+^, 0.3% bovine serum albumin (BSA), and 1% P/S. After 1 h at 37°C, the inoculum was removed, cells were washed once with PBS, and postinfection medium was added: DMEM supplemented with 0.1% FBS, 0.3% BSA, 20 nM HEPES, 1% P/S, and 1 μg/ml TPCK-treated trypsin, together with 6 μM *Renilla* luciferase substrate (EnduRen live-cell substrate; Promega). Where noted, the virus inoculum was preincubated with the indicated concentrations of monoclonal anti-H5 antibody (no. 200-301-976; Rockland) or polyclonal anti-H5 serum (no. 2705; BEI Resources) at 4°C for 1 h before infection of cells. Also where noted, the indicated concentrations of baloxavir acid (no. HY-109025A; MedChemExpress) or S20 fusion inhibitor (58) (ChemBridge) were added to the cells following infection. Real-time luminescence measurements were taken at various time points using an EnVision multilabel reader (Perkin Elmer) or a Dynex MLX luminometer (Dynex Technologies). All experiments included infection of multiple wells for technical replicates, and each experiment was independently performed at least 3 times. Mock-infected wells, otherwise treated identically, acted as negative controls for background luminescence from the reagents.

### *In vitro* NA activity assay.

To measure NA activity of virus preparations, the NA-Star influenza virus neuraminidase inhibitor resistance detection kit (Applied Biosystems) was used according to the manufacturer’s protocol. As necessary, virus preparations were pretreated for 1 h with various concentrations of oseltamivir carboxylate (no. sc-212484; Santa Cruz Biotechnology) or zanamivir (no. SML0492; Sigma-Aldrich), and chemiluminescence was measured with the EnVision multilabel reader (Perkin Elmer).

### Data analysis.

For *Renilla* luciferase assays, raw luminescence values were averaged from technical replicates within the same experiment, normalized to mock (background) values, and made relative to the initial value of the MOI = 1 condition. Each independent replicate was processed separately. Where necessary, the area under the curve (AUC) of the replication kinetics was determined and used to calculate half-maximal inhibitory concentrations (IC_50_) from the resulting nonlinear regression curve. For analysis of the NA activity assay, raw luminescence values were normalized to background, averaged, and made relative to the untreated control. The IC_50_ was calculated from the resulting nonlinear regression curve. These analyses, and all statistics, were performed using GraphPad Prism version 7.05.

## References

[1] WHO. 2018. Influenza (seasonal) factsheet. https://www.who.int/news-room/fact-sheets/detail/influenza-(seasonal). Accessed 4 January 2020.

[2] WHO. 2018. Influenza (avian and other zoonotic) factsheet. https://www.who.int/news-room/fact-sheets/detail/influenza-(avian-and-other-zoonotic). Accessed 4 January 2020.

[3] Horimoto T, Kawaoka Y. 2005. Influenza: lessons from past pandemics, warnings from current incidents. Nat Rev Microbiol 3:591–600. doi:10.1038/nrmicro1208. 16064053

[4] Krammer F, Smith GJD, Fouchier RAM, Peiris M, Kedzierska K, Doherty PC, Palese P, Shaw ML, Treanor J, Webster RG, Garcia-Sastre A. 2018. Influenza. Nat Rev Dis Primers 4:3. doi:10.1038/s41572-018-0002-y. 29955068 PMC7097467

[5] Viboud C, Simonsen L, Fuentes R, Flores J, Miller MA, Chowell G. 2016. Global mortality impact of the 1957–1959 influenza pandemic. J Infect Dis 213:738–745. doi:10.1093/infdis/jiv534. 26908781 PMC4747626

[6] Jester BJ, Uyeki TM, Jernigan DB. 2020. Fifty years of influenza A(H3N2) following the pandemic of 1968. Am J Public Health 110:669–676. doi:10.2105/AJPH.2019.305557. 32267748 PMC7144439

[7] Viboud C, Grais RF, Lafont BA, Miller MA, Simonsen L, Multinational Influenza Seasonal Mortality Study Group. 2005. Multinational impact of the 1968 Hong Kong influenza pandemic: evidence for a smoldering pandemic. J Infect Dis 192:233–248. doi:10.1086/431150. 15962218

[8] Davis CT, Chen LM, Pappas C, Stevens J, Tumpey TM, Gubareva LV, Katz JM, Villanueva JM, Donis RO, Cox NJ. 2014. Use of highly pathogenic avian influenza A(H5N1) gain-of-function studies for molecular-based surveillance and pandemic preparedness. mBio 5:e02431-14. doi:10.1128/mBio.02431-14. 25505125 PMC4278543

[9] Schultz-Cherry S, Webby RJ, Webster RG, Kelso A, Barr IG, McCauley JW, Daniels RS, Wang D, Shu Y, Nobusawa E, Itamura S, Tashiro M, Harada Y, Watanabe S, Odagiri T, Ye Z, Grohmann G, Harvey R, Engelhardt O, Smith D, Hamilton K, Claes F, Dauphin G. 2014. Influenza gain-of-function experiments: their role in vaccine virus recommendation and pandemic preparedness. mBio 5:e02430-14. doi:10.1128/mBio.02430-14. 25505124 PMC4278542

[10] Herfst S, Schrauwen EJ, Linster M, Chutinimitkul S, de Wit E, Munster VJ, Sorrell EM, Bestebroer TM, Burke DF, Smith DJ, Rimmelzwaan GF, Osterhaus AD, Fouchier RA. 2012. Airborne transmission of influenza A/H5N1 virus between ferrets. Science 336:1534–1541. doi:10.1126/science.1213362. 22723413 PMC4810786

[11] Imai M, Watanabe T, Hatta M, Das SC, Ozawa M, Shinya K, Zhong G, Hanson A, Katsura H, Watanabe S, Li C, Kawakami E, Yamada S, Kiso M, Suzuki Y, Maher EA, Neumann G, Kawaoka Y. 2012. Experimental adaptation of an influenza H5 HA confers respiratory droplet transmission to a reassortant H5 HA/H1N1 virus in ferrets. Nature 486:420–428. doi:10.1038/nature10831. 22722205 PMC3388103

[12] Tumpey TM, Basler CF, Aguilar PV, Zeng H, Solorzano A, Swayne DE, Cox NJ, Katz JM, Taubenberger JK, Palese P, Garcia-Sastre A. 2005. Characterization of the reconstructed 1918 Spanish influenza pandemic virus. Science 310:77–80. doi:10.1126/science.1119392. 16210530

[13] Tumpey TM, Maines TR, Van Hoeven N, Glaser L, Solorzano A, Pappas C, Cox NJ, Swayne DE, Palese P, Katz JM, Garcia-Sastre A. 2007. A two-amino acid change in the hemagglutinin of the 1918 influenza virus abolishes transmission. Science 315:655–659. doi:10.1126/science.1136212. 17272724

[14] Fouchier RA, Kawaoka Y, Cardona C, Compans RW, Garcia-Sastre A, Govorkova EA, Guan Y, Herfst S, Orenstein WA, Peiris JS, Perez DR, Richt JA, Russell C, Schultz-Cherry SL, Smith DJ, Steel J, Tompkins SM, Topham DJ, Treanor JJ, Tripp RA, Webby RJ, Webster RG. 2013. Gain-of-function experiments on H7N9. Science 341:612–613. doi:10.1126/science.341.6146.612. 23929965

[15] Rey F, Schwartz O, Wain-Hobson S. 2013. Gain-of-function research: unknown risks. Science 342:311. doi:10.1126/science.342.6156.311-a. 24136951

[16] Lipsitch M. 2018. Why do exceptionally dangerous gain-of-function experiments in influenza? Methods Mol Biol 1836:589–608. doi:10.1007/978-1-4939-8678-1_29. 30151594 PMC7119956

[17] Imperiale MJ, Howard D, Casadevall A. 2018. The silver lining in gain-of-function experiments with pathogens of pandemic potential. Methods Mol Biol 1836:575–587. doi:10.1007/978-1-4939-8678-1_28. 30151593 PMC7120448

[18] Imperiale MJ, Casadevall A. 2018. A new approach to evaluating the risk-benefit equation for dual-use and gain-of-function research of concern. Front Bioeng Biotechnol 6:21. doi:10.3389/fbioe.2018.00021. 29568736 PMC5853790

[19] Adam DC, Magee D, Bui CM, Scotch M, MacIntyre CR. 2017. Does influenza pandemic preparedness and mitigation require gain-of-function research? Influenza Other Respir Viruses 11:306–310. doi:10.1111/irv.12458. 28502086 PMC5485867

[20] Evans NG, Lipsitch M, Levinson M. 2015. The ethics of biosafety considerations in gain-of-function research resulting in the creation of potential pandemic pathogens. J Med Ethics 41:901–908. doi:10.1136/medethics-2014-102619. 26320212 PMC4623968

[21] Duprex WP, Fouchier RA, Imperiale MJ, Lipsitch M, Relman DA. 2015. Gain-of-function experiments: time for a real debate. Nat Rev Microbiol 13:58–64. doi:10.1038/nrmicro3405. 25482289 PMC7097416

[22] Casadevall A, Howard D, Imperiale MJ. 2014. The apocalypse as a rhetorical device in the influenza virus gain-of-function debate. mBio 5:e02062-14. doi:10.1128/mBio.02062-14. 25316704 PMC4205799

[23] Casadevall A, Howard D, Imperiale MJ. 2014. An epistemological perspective on the value of gain-of-function experiments involving pathogens with pandemic potential. mBio 5:e01875-14. doi:10.1128/mBio.01875-14. 25227471 PMC4172079

[24] Casadevall A, Imperiale MJ. 2014. Risks and benefits of gain-of-function experiments with pathogens of pandemic potential, such as influenza virus: a call for a science-based discussion. mBio 5:e01730-14. doi:10.1128/mBio.01730-14. 25085113 PMC4128368

[25] Wain-Hobson S. 2014. The irrationality of GOF avian influenza virus research. Front Public Health 2:77. doi:10.3389/fpubh.2014.00077. 25077136 PMC4099557

[26] Wain-Hobson S. 2013. Pandemic influenza viruses: time to recognize our inability to predict the unpredictable and stop dangerous gain-of-function experiments. EMBO Mol Med 5:1637–1641. doi:10.1002/emmm.201303475. 24186378 PMC3840482

[27] Fouchier RA. 2015. Studies on influenza virus transmission between ferrets: the public health risks revisited. mBio 6:e02560-14. doi:10.1128/mBio.02560-14. 25616377 PMC4323420

[28] Klotz LC. 2015. Danger of potential-pandemic-pathogen research enterprises. mBio 6:e00815-15. doi:10.1128/mBio.00815-15. 26081636 PMC4471565

[29] Klotz LC. 2015. Comments on Fouchier's calculation of risk and elapsed time for escape of a laboratory-acquired infection from his laboratory. mBio 6:e00268-15. doi:10.1128/mBio.00268-15. 25873376 PMC4453553

[30] Fouchier RA. 2015. Reply to “Comments on Fouchier's calculation of risk and elapsed time for escape of a laboratory-acquired infection from his laboratory.” mBio 6:e00407-15. doi:10.1128/mBio.00407-15. 25873379 PMC4453508

[31] Lipsitch M. 2014. Can limited scientific value of potential pandemic pathogen experiments justify the risks? mBio 5:e02008-14. doi:10.1128/mBio.02008-14. 25316701 PMC4205796

[32] Lipsitch M, Inglesby TV. 2015. Reply to “Studies on influenza virus transmission between ferrets: the public health risks revisited.” mBio 6:e00041-15. doi:10.1128/mBio.00041-15. 25616376 PMC4323416

[33] Fouchier RA, Garcia-Sastre A, Kawaoka Y, Barclay WS, Bouvier NM, Brown IH, Capua I, Chen H, Compans RW, Couch RB, Cox NJ, Doherty PC, Donis RO, Feldmann H, Guan Y, Katz JM, Kiselev OI, Klenk HD, Kobinger G, Liu J, Liu X, Lowen A, Mettenleiter TC, Osterhaus AD, Palese P, Peiris JS, Perez DR, Richt JA, Schultz-Cherry S, Steel J, Subbarao K, Swayne DE, Takimoto T, Tashiro M, Taubenberger JK, Thomas PG, Tripp RA, Tumpey TM, Webby RJ, Webster RG. 2013. Transmission studies resume for avian flu. Science 339:520–521. doi:10.1126/science.1235140. 23345603 PMC3838856

[34] Fouchier RA, Garcia-Sastre A, Kawaoka Y, Barclay WS, Bouvier NM, Brown IH, Capua I, Chen H, Compans RW, Couch RB, Cox NJ, Doherty PC, Donis RO, Feldmann H, Guan Y, Katz J, Klenk HD, Kobinger G, Liu J, Liu X, Lowen A, Mettenleiter TC, Osterhaus AD, Palese P, Peiris JS, Perez DR, Richt JA, Schultz-Cherry S, Steel J, Subbarao K, Swayne DE, Takimoto T, Tashiro M, Taubenberger JK, Thomas PG, Tripp RA, Tumpey TM, Webby RJ, Webster RG. 2012. Pause on avian flu transmission research. Science 335:400–401. doi:10.1126/science.335.6067.400. 22282787 PMC3812248

[35] Fouchier RA, Garcia-Sastre A, Kawaoka Y. 2012. Pause on avian flu transmission studies. Nature 481:443. doi:10.1038/481443a. 22266939

[36] Lipsitch M, Inglesby TV. 2014. Moratorium on research intended to create novel potential pandemic pathogens. mBio 5:e02366-14. doi:10.1128/mBio.02366-14. 25505122 PMC4271556

[37] Fears R, ter Meulen V. 2015. European academies advise on gain-of-function studies in influenza virus research. J Virol 90:2162–2164. doi:10.1128/JVI.03045-15. 26699646 PMC4810713

[38] Casadevall A, Dermody TS, Imperiale MJ, Sandri-Goldin RM, Shenk T. 2014. On the need for a national board to assess dual use research of concern. J Virol 88:6535–6537. doi:10.1128/JVI.00875-14. 24696484 PMC4054389

[39] Lipkin WI. 2012. Biocontainment in gain-of-function infectious disease research. mBio 3:e00290-12. doi:10.1128/mBio.00290-12. PMC3484385 23047747

[40] Fouchier RA, Garcia-Sastre A, Kawaoka Y. 2012. The pause on Avian H5N1 influenza virus transmission research should be ended. mBio 3:e00358-12. doi:10.1128/mBio.00358-12. 23047750 PMC3484389

[41] Garcia-Sastre A. 2012. Working safely with H5N1 viruses. mBio 3:e00049-12. doi:10.1128/mBio.00049-12. 22396483 PMC3302572

[42] Halfmann P, Kim JH, Ebihara H, Noda T, Neumann G, Feldmann H, Kawaoka Y. 2008. Generation of biologically contained Ebola viruses. Proc Natl Acad Sci U S A 105:1129–1133. doi:10.1073/pnas.0708057105. 18212124 PMC2234103

[43] Zhang Q, Li N, Deng C, Zhang Z, Li X, Yoshii K, Ye H, Zhang B. 2019. Trans complementation of replication-defective Omsk hemorrhagic fever virus for antiviral study. Virol Sin 34:412–422. doi:10.1007/s12250-019-00109-0. 30949960 PMC6687815

[44] Nogales A, Baker SF, Domm W, Martinez-Sobrido L. 2016. Development and applications of single-cycle infectious influenza A virus (sciIAV). Virus Res 216:26–40. doi:10.1016/j.virusres.2015.07.013. 26220478 PMC4728073

[45] Marsh GA, Hatami R, Palese P. 2007. Specific residues of the influenza A virus hemagglutinin viral RNA are important for efficient packaging into budding virions. J Virol 81:9727–9736. doi:10.1128/JVI.01144-07. 17634232 PMC2045411

[46] Konig R, Stertz S, Zhou Y, Inoue A, Hoffmann HH, Bhattacharyya S, Alamares JG, Tscherne DM, Ortigoza MB, Liang Y, Gao Q, Andrews SE, Bandyopadhyay S, De Jesus P, Tu BP, Pache L, Shih C, Orth A, Bonamy G, Miraglia L, Ideker T, Garcia-Sastre A, Young JA, Palese P, Shaw ML, Chanda SK. 2010. Human host factors required for influenza virus replication. Nature 463:813–817. doi:10.1038/nature08699. 20027183 PMC2862546

[47] Steel J, Lowen AC, Pena L, Angel M, Solorzano A, Albrecht R, Perez DR, Garcia-Sastre A, Palese P. 2009. Live attenuated influenza viruses containing NS1 truncations as vaccine candidates against H5N1 highly pathogenic avian influenza. J Virol 83:1742–1753. doi:10.1128/JVI.01920-08. 19073731 PMC2643794

[48] Close DM, Hahn RE, Patterson SS, Baek SJ, Ripp SA, Sayler GS. 2011. Comparison of human optimized bacterial luciferase, firefly luciferase, and green fluorescent protein for continuous imaging of cell culture and animal models. J Biomed Opt 16:e047003. doi:10.1117/1.3564910. PMC3094131 21529093

[49] O'Hanlon R, Shaw ML. 2019. Baloxavir marboxil: the new influenza drug on the market. Curr Opin Virol 35:14–18. doi:10.1016/j.coviro.2019.01.006. 30852344

[50] Takashita E, Morita H, Ogawa R, Nakamura K, Fujisaki S, Shirakura M, Kuwahara T, Kishida N, Watanabe S, Odagiri T. 2018. Susceptibility of influenza viruses to the novel cap-dependent endonuclease inhibitor baloxavir marboxil. Front Microbiol 9:3026. doi:10.3389/fmicb.2018.03026. 30574137 PMC6291754

[51] Noshi T, Kitano M, Taniguchi K, Yamamoto A, Omoto S, Baba K, Hashimoto T, Ishida K, Kushima Y, Hattori K, Kawai M, Yoshida R, Kobayashi M, Yoshinaga T, Sato A, Okamatsu M, Sakoda Y, Kida H, Shishido T, Naito A. 2018. In vitro characterization of baloxavir acid, a first-in-class cap-dependent endonuclease inhibitor of the influenza virus polymerase PA subunit. Antiviral Res 160:109–117. doi:10.1016/j.antiviral.2018.10.008. 30316915

[52] Langlois RA, Albrecht RA, Kimble B, Sutton T, Shapiro JS, Finch C, Angel M, Chua MA, Gonzalez-Reiche AS, Xu K, Perez D, Garcia-Sastre A, tenOever BR. 2013. MicroRNA-based strategy to mitigate the risk of gain-of-function influenza studies. Nat Biotechnol 31:844–847. doi:10.1038/nbt.2666. 23934176 PMC3808852

[53] de Vries RP, Zhu X, McBride R, Rigter A, Hanson A, Zhong G, Hatta M, Xu R, Yu W, Kawaoka Y, de Haan CA, Wilson IA, Paulson JC. 2014. Hemagglutinin receptor specificity and structural analyses of respiratory droplet-transmissible H5N1 viruses. J Virol 88:768–773. doi:10.1128/JVI.02690-13. 24173215 PMC3911709

[54] Chutinimitkul S, van Riel D, Munster VJ, van den Brand JM, Rimmelzwaan GF, Kuiken T, Osterhaus AD, Fouchier RA, de Wit E. 2010. In vitro assessment of attachment pattern and replication efficiency of H5N1 influenza A viruses with altered receptor specificity. J Virol 84:6825–6833. doi:10.1128/JVI.02737-09. 20392847 PMC2903244

[55] Stevens J, Blixt O, Glaser L, Taubenberger JK, Palese P, Paulson JC, Wilson IA. 2006. Glycan microarray analysis of the hemagglutinins from modern and pandemic influenza viruses reveals different receptor specificities. J Mol Biol 355:1143–1155. doi:10.1016/j.jmb.2005.11.002. 16343533

[56] Zhang Y, Zhang Q, Kong H, Jiang Y, Gao Y, Deng G, Shi J, Tian G, Liu L, Liu J, Guan Y, Bu Z, Chen H. 2013. H5N1 hybrid viruses bearing 2009/H1N1 virus genes transmit in guinea pigs by respiratory droplet. Science 340:1459–1463. doi:10.1126/science.1229455. 23641061

[57] Xiong X, Coombs PJ, Martin SR, Liu J, Xiao H, McCauley JW, Locher K, Walker PA, Collins PJ, Kawaoka Y, Skehel JJ, Gamblin SJ. 2013. Receptor binding by a ferret-transmissible H5 avian influenza virus. Nature 497:392–396. doi:10.1038/nature12144. 23615615

[58] White KM, De Jesus P, Chen Z, Abreu P, Jr, Barile E, Mak PA, Anderson P, Nguyen QT, Inoue A, Stertz S, Koenig R, Pellecchia M, Palese P, Kuhen K, Garcia-Sastre A, Chanda SK, Shaw ML. 2015. A potent anti-influenza compound blocks fusion through stabilization of the prefusion conformation of the hemagglutinin protein. ACS Infect Dis 1:98–109. doi:10.1021/id500022h. 25984567 PMC4426349

[59] Nohinek B, Gerhard W, Schulze IT. 1985. Characterization of host cell binding variants of influenza virus by monoclonal antibodies. Virology 143:651–656. doi:10.1016/0042-6822(85)90407-6. 2414912

[60] Manicassamy B, Medina RA, Hai R, Tsibane T, Stertz S, Nistal-Villan E, Palese P, Basler CF, Garcia-Sastre A. 2010. Protection of mice against lethal challenge with 2009 H1N1 influenza A virus by 1918-like and classical swine H1N1 based vaccines. PLoS Pathog 6:e1000745. doi:10.1371/journal.ppat.1000745. 20126449 PMC2813279

[61] Fodor E, Devenish L, Engelhardt OG, Palese P, Brownlee GG, Garcia-Sastre A. 1999. Rescue of influenza A virus from recombinant DNA. J Virol 73:9679–9682. doi:10.1128/JVI.73.11.9679-9682.1999. 10516084 PMC113010

[62] Medina RA, Stertz S, Manicassamy B, Zimmermann P, Sun X, Albrecht RA, Uusi-Kerttula H, Zagordi O, Belshe RB, Frey SE, Tumpey TM, Garcia-Sastre A. 2013. Glycosylations in the globular head of the hemagglutinin protein modulate the virulence and antigenic properties of the H1N1 influenza viruses. Sci Transl Med 5:187ra70. doi:10.1126/scitranslmed.3005996. PMC3940933 23720581

[63] McGinnis J, Laplante J, Shudt M, George KS. 2016. Next generation sequencing for whole genome analysis and surveillance of influenza A viruses. J Clin Virol 79:44–50. doi:10.1016/j.jcv.2016.03.005. 27085509

[64] Zhou B, Donnelly ME, Scholes DT, St George K, Hatta M, Kawaoka Y, Wentworth DE. 2009. Single-reaction genomic amplification accelerates sequencing and vaccine production for classical and Swine origin human influenza A viruses. J Virol 83:10309–10313. doi:10.1128/JVI.01109-09. 19605485 PMC2748056

